# Social isolation, perceived social support, and type D personality among gastrointestinal cancer survivors in China: A mediating model analysis

**DOI:** 10.1016/j.apjon.2024.100617

**Published:** 2024-11-09

**Authors:** Yuqiu Zhao, Yeming Xu, Tianxiu Wang, Mingbo Hua, Shantanu Baral, Qiannan Sun, Daorong Wang

**Affiliations:** a Yangzhou University School of Nursing School of Public Health, Yangzhou, China; b Northern Jiangsu People's Hospital, Yangzhou, China; c General Surgery Institute of Yangzhou, Yangzhou University, Yangzhou, China; d Yangzhou Key Laboratory of Basic and Clinical Transformation of Digestive and Metabolic Diseases, Yangzhou, China; e Yangzhou University Medical College, Yangzhou University, Yangzhou, China

**Keywords:** Gastrointestinal cancer survivors, Social isolation, Perceived social support, Type D personality, Mediating effects

## Abstract

**Objective:**

To explore the mediating effect of social isolation between perceived social support and type D personality in gastrointestinal cancer survivors based on the WHITE heuristic cognitive-behavioral model of emotion cognition theory.

**Methods:**

The study utilized a convenience sampling method to recruit 183 survivors of gastrointestinal cancer who were undergoing chemoradiotherapy. SPSS 26.0 was used to analyze the correlation among social isolation, perceived social support, and type D personality. Additionally, SmartPLS 3.0 was utilized to analyze the mediating role of social isolation in the relationships between perceived social support and type D personality.

**Results:**

The study found that gastrointestinal cancer survivors scored 60.58 ​± ​10.94 for perceived social support, 36.25 ​± ​4.71 for social isolation, and 26.26 ​± ​5.84 for type D personality. We discovered that perceived social support has a negative correlation with social isolation and type D personality (*r* ​= ​−0.593, −0.396, both *P* ​< ​0.001), while social isolation was positively correlated with type D personality (*r* ​= ​0.564, *P* ​< ​0.001). The association between type D personality and perceived social support was partially mediated by social isolation (VAF ​= ​59.67%).

**Conclusions:**

The association between type D personality and perceived social support is mediated by social isolation in gastrointestinal cancer survivors undergoing chemoradiotherapy. Clinical staff should prioritize evaluating social isolation among these survivors to enhance social support and potentially reduce the prevalence of type D personality traits.

## Introduction

According to the latest global cancer report for 2020 released by the World Health Organisation's International Agency for Research on Cancer (IARC), in China, there were 4.57 million new cancer cases and 3 million deaths, contributing significantly to the global burden of cancer. Gastric and colorectal cancers are among the top five in terms of new incidence and mortality rates globally, as well as in China.^1^ With the gradual implementation of cancer screening, more patients can be diagnosed early. Concurrently, advancements in medical technology have improved the survival rate of cancer patients, resulting in a large and special group of cancer survivors.^2^

At present, the number of gastrointestinal cancer survivors is growing, and the quality of their survival is also attracting more and more attention. Psychosocial adaptation and social integration of cancer survivors are important indicators of their quality of survival.^3^ Research shows that the main clinical treatment for gastrointestinal cancer patients is surgery combined with chemotherapy and radiotherapy. While chemotherapy and radiotherapy can extend a patient's life, they simultaneously target cancer cells and lead to a range of side effects, including fatigue, hair loss, nausea, vomiting, bone marrow suppression, fear, and depression.^4^, ^5^, ^6^

In addition, for patients with enterostomies, the prolonged presence of the stoma is an intense stressful event for the patient. Because of the inability to autonomously control defecation, the sound of stoma exhaust, the stoma bag attached to the abdominal wall, the mucosa of the intestinal tube exposed and other factors make the patient's postoperative image disordered, the patient's poor postoperative self-care is prone to cause irritant dermatitis, parastomal hernia, and other complications, coupled with the long-term consumption of stoma care supplies, increasing the cost of family expenditure, leading to the patient's anxiety, avoidance, low self-esteem, and other distressing emotions.^7^^,^^8^ The powerful physical and mental double stress causes patients to have a serious sense of shame and may gradually detach themselves from the social group of social isolation problems.^9^ Social isolation in adults with cancer refers to individuals exhibiting behaviors and states of social avoidance, such as avoiding contact with others and concealing their emotions, as well as experiencing distinct negative affective states, including feelings of loneliness.^10^ Relevant studies have shown that social isolation not only aggravates the physical, psychological, and social burdens of cancer survivors, but also leads to nutritional risks, reduced survival rates, and serious adverse consequences for patients.^11^^,^^12^ Attention to the social isolation of cancer patients is of great significance to promote the return of patients to their families and society and to improve the quality of survival.

With the deepening of the physiological-psychological-social medical model, improving patients' mental health has gradually become a hot topic of concern for clinical healthcare professionals. Focusing on the social isolation level of gastrointestinal cancer survivors of postoperative chemoradiotherapy is of great significance in reducing individual psychological pain, increasing social integration, and obtaining ideal rehabilitation outcomes.^13^

At present, studies on social isolation in China mainly focus on migrant populations, the elderly, adolescents, certain special groups, and groups of cancer patients, and research on the social isolation status of cancer survivors is still in the initial stage, and few studies on social isolation of gastrointestinal cancer survivors have been reported. Gastrointestinal cancer survivors will face a series of social problems such as marriage and childbearing, employment, returning to work, and old age during their survival period, but their competitiveness is at a disadvantage due to health problems, and this, coupled with the influence of negative psychology such as low self-esteem and disease stigma, is highly likely to lead to social isolation. Therefore, we should pay attention to the social isolation of gastrointestinal cancer survivors and the factors affecting it, and actively intervene to promote their social integration.

A depressed personality with high degrees of social inhibition (SI) and negative affectivity (NA) is referred to as a type D personality, including nervousness, unhappiness, excessive worry, pessimism, and irritability, along with social withdrawal.^14^ In the field of oncology, little research has been conducted on type D personality. Existing studies have focused on colorectal cancer survivors, 19% of whom can be categorized as type D personalities.^15^ These patients have more comorbidities and greater healthcare utilization.^16^

A review written by Denollet et al. concluded that type D personality is associated with the level/frequency of suicidal ideation.^17^ A recent study of gastric cancer survivors in China showed a statistically significant difference in three-year overall survival between those with and without type D personalities.^18^ Mols et al.^19^ found that type D personality is a stable^20^ and powerful predictor of impaired quality of life and mental health status.

Hao et al.^21^ found that social isolation was associated with type D personality in a study of patients undergoing postoperative chemotherapy for ovarian cancer, and showed that this socially inhibited trait would lead to difficulties in obtaining adequate social support for depression, internalization of repressed emotions, and reduced social functioning. There is an association between type D personality and social isolation in patients with cardiovascular disease and the general population. For example, Michal et al.^22^ showed that individuals with type D personality were more likely to experience social isolation in patients with cardiovascular disease and the general population, and Lee et al. showed in a study of diabetic patients that type D personality indirectly affects glycosylated hemoglobin (HbA1c) and health-related quality of life (HRQoL) through diabetic distress and social isolation.^23^

Type D personality is a hot research topic in cardiovascular disease, and there have been some recent studies on the general population, and chronic diseases, with little reported in the population of cancer patients. However, at present, the research studying type D personality in cancer patients is a foreign population, and within China, the research on type D personality in cancer survivors has just begun. Therefore, there is a need to investigate the factors associated with type D personality and to mitigate the adverse emotional effects of type D personality in the general population through individualized interventions.

Perceived social support is an individual's emotional experience and the level of satisfaction they have when they feel respected, understood, and supported within society.^24^ Previous studies have shown that perceived social support is one of the influential factors in improving negative emotions such as anxiety, depression, and loneliness in cancer survivors.^25^^,^^26^ Specifically, good social interactions can provide cancer survivors with emotional support and protect them from psychological problems. The review by Zhang et al. concluded that social support is an important predictor of loneliness and that higher levels of social support are negatively correlated with loneliness.^27^ The results of this study support the strong link between social support and loneliness and emphasize the important role of social support in reducing levels of loneliness. Some relevant studies have indicated that perceived social support plays a partial mediating role in the relationship between loneliness and anxiety, depression, and somatic symptoms.^28^ Currently, research on the relationship between perceived social support and social detachment has focused on older populations and less on gastrointestinal populations, so attention needs to be paid to the relationship between perceived social support and social detachment in gastrointestinal cancer populations.

The WHITE^29^ heuristic cognitive-behavioral model of emotional cognition theory, suggests that when patients experience somatic dysfunction and perceive insufficient social support, they tend to socially detach and develop persistent negative emotions. According to this model, the negative emotions resulting from social isolation may stem from perceived inadequate social support following changes in bodily functioning. The association between perceived social support, and social isolation, and their impact on type D personality among gastrointestinal cancer survivors is currently unclear in research studies. This study investigates the relationship between perceived social support, social isolation, and type D personality in gastrointestinal cancer survivors and explores the mediating effect of social isolation between perceived social support and type D personality, aiming to provide a basis for reducing the prevalence of type D personality in this cancer survivors, to alleviate dysphoria in gastrointestinal cancer survivors with type D personalities.

Based on the literature review and the above theoretical considerations, this study makes the following research hypotheses: Hypothesis 1: There is a significant correlation between social isolation and comprehension social support and type D personality in gastrointestinal cancer survivors, and there is a certain path relationship among the three; Hypothesis 2: Social isolation mediates the relationship between comprehension social support and type D personality.

## Methods

### Study design, setting, and participants

Patients with gastrointestinal cancer attending the Department of Gastrointestinal Surgery at a tertiary-level hospital in Yangzhou City, Jiangsu Province, from October 2023 to June 2024 were selected for the study using a convenience sampling method. Inclusion criteria: (1) Diagnosed with a gastrointestinal malignant tumor by preoperative pathology; (2) Aged 18 years or older; (3) Radiotherapy, chemotherapy, and other treatments are ongoing; (4) Clear thinking, normal hearing, and language expression; (5) Aware of their condition and willing to cooperate with this study. Exclusion criteria: (1) Presence of cognitive impairment or other psychiatric disorders; (2) Severe primary diseases, such as heart, brain, and kidneys, and severe organ failure.

In this study, GPower 3.1 served as a pivotal tool for estimating sample size, employing correlation analysis as the statistical backbone. By adhering to the medium effect sizes of f ​= ​0.30, *α* ​= ​0.05, and 1 - *β* ​= ​0.95, a minimum total sample size of 134 cases was established. To further refine this estimation, categorical regression analyses were conducted, encompassing main effects and dichotomous interaction effects, alongside mediational analyses that required medium effect sizes of f^2^ ​= ​0.15, *α* ​= ​0.05, and 1 - *β* ​= ​0.80. The study incorporated nine predictors—two for type D personality, four for social isolation, and three for perceived social support—culminating in a necessary minimum sample size of 114. Ultimately, the larger figure of 134 cases was adopted as the definitive minimum sample size for the research, ensuring robust statistical integrity throughout the investigation.

### Measurements

#### Sociodemographic and cancer characteristics

The questionnaire was compiled by the researcher based on extensive literature and included two parts: demographic information and disease-related information.

The demographic data included age, gender, education level, body mass index (BMI), marital status, residence status, per capita monthly household income, and more. The disease-related data included disease type, inflammatory factors, and other relevant information. The questionnaire was meticulously designed by the researcher, drawing on a wealth of literature.

#### Social isolation

The general alienation scale (GAS) was developed by Jessor et al.^30^ to evaluate college students' sense of alienation from their surroundings, with a scale Cronbach's alpha coefficient ranging from 0.812 to 0.879. It was translated and revised by Wu Shuang et al.^31^ in 2015 to measure the sense of social alienation in older adults, yielding a total Cronbach's alpha coefficient of 0.770.

The scale includes 15 items and 4 dimensions: self-alienation (3 items), others' alienation (5 items), sense of doubt (4 items), and sense of meaninglessness (3 items). ranging from 1 (strongly disagree) to 4 (strongly agree), with total scores ranging from 15 to 60. Higher scores signify higher social isolation. The Cronbach's alpha coefficient for this scale in our study was 0.878.

#### Perceived social support

Perceived Social Support Scale (PSSS) was developed by Zimet^32^ in 1987, the Scale (PSSS) is mainly used to measure the degree of social support perceived by individuals from family, friends, and others. The PSSS comprises 12 items, rated on a seven-point Likert scale from “strongly disagree” (1) to “strongly agree” (7). Total scores range from 12 to 84. There are two ways of dividing the dimensions of the scale: (1) Dividing the scale into three dimensions: family support, friend support, and other support; (2) Dividing the scale into two dimensions: intra-family support and extra-family support. This study used the first-dimension division criterion. The scale has been widely used in both domestic and international studies. The internal consistency reliability coefficient of the PSSS scale in this study was 0.926.

#### Type D personality

Type D Personality Scale-14 (DS14) was prepared by Dutch scholar Denollet^20^ in 2005, the type D Personality Scale includes 2 dimensions (7 items each) of social inhibition and negative affect. The social inhibition dimension includes questions 1, 3, 6, 8, 10, 11, and 14, while the negative affect dimension includes questions 2, 4, 5, 7, 9, 12, and 13. Ranging from “very non-conforming” (0 points) to “very conforming” (4 points). The criteria for assessing type D personality are based on a cut-off score of 10 for both dimensions. Specifically, a person must score ≥ 10 on both the negative affect (NA) and social inhibition (SI) dimensions to be recognized as having a type D personality.

### Data collection

The questionnaire method was used and was administered by the researcher personally. Firstly, the purpose and significance of the study were elucidated to gastrointestinal cancer survivors who fulfilled the inclusion criteria through uniform instructions. The questionnaire was distributed after obtaining informed consent. The questionnaires were filled out anonymously, Collected on-site, and verified for accuracy. If there were omissions or incorrect options, the researcher guided the completion, avoiding the use of leading language. Out of 200 questionnaires distributed, 183 were considered valid as those with identical answers or repeated patterns were excluded. This yielded an effective recovery rate of 91.5%.

### Data analysis

IBM SPSS 26.0 and SmartPLS 3.0 were used to analyze the data. Count data were described by frequency (f) and percentage; measure data were described by (Mean ± SD) and M (P25, P75); The Mann–Whitney U test or Kruskal–Wallis test was performed with the patient's type D personality total score as the dependent variable and the patient's general demographic information as the independent variable. Spearman rank correlation analysis was used to test the correlation between social isolation, perceived social support, and type D personality; Least squares method (LES) was used to establish SEM and validate it to explore the mediating effect of social isolation between perceived social support and type D personality. The SEM established by LES consists of 2 parts: measurement model and structural model. The evaluation indexes of the measurement model are reliability and validity. Reliability includes Cronbach's *α* (CA) reliability and combined reliability (CR); validity includes convergent validity and discriminant validity, the former is reflected by factor loadings, average extracted variance (AVE), and reliability; CA, CR, factor loadings > 0.7, and AVE > 0.5 represent better convergent validity,^33^ a greater square root of the average value (AVE) of each latent variable compared to its correlation coefficient with other latent variables signifies stronger discriminant validity.^34^ The evaluation of the structural model includes three aspects: path analysis, explained variance value (*R*^2^) and goodness of fit (GOF) of the model. The results of path analysis are mainly reflected by the path coefficient, t-value and *P*-value,^35^ with *P* ​< ​0.05 as the difference is statistically significant; *R*^2^ ​≥ ​0.190 indicates that the model meets the requirements;^34^ GOF represents the overall predictive power of the model, which is given by GOF ​= ​$\sqrt{\bar{Communality}*\bar{R^{2}}}$, with GOF ​= ​0.100 representing a small predictive power of the model, GOF ​= ​0.250 representing a moderate predictive power of the model, and GOF ​= ​0.360 representing a large predictive power.^33^

### Ethical considerations

This study has been approved by the Ethics Committee of Yangzhou University School of Nursing School of Public Health (IRB No. YZUHL20230078). All participants provided written informed consent.

## Results

### Descriptive statistics of participants’ characteristics

There were 183 gastrointestinal cancer survivors in the study. The patients' ages ranged from 32 to 89 years, with a mean age of 67.89 ​± ​9.73 years. The BMI ranged from 17.10 to 31.09 kg/m^2^, with a mean of 22.93 ​± ​2.78 kg/m^2^. Among colorectal cancer survivors, 10 (5.5%) carry a colostomy. The Mann–Whitney U test or Kruskal–Wallis test was performed with the patient's total type D personality score as the dependent variable and the patients' general demographic information as the independent variable. The results showed that there was a statistically significant difference in the type D personality scores of patients with different residency statuses (*P* ​< ​0.05), and there was no statistically significant difference between the other factors and the total type D personality score (*P* ​> ​0.05). Other general information is shown in Table 1.

**Table 1:** General information on gastrointestinal cancer survivors (*N* ​= ​183).

Variables	Characteristics	*n* (%)	Total score of type D personality [M (P25, P75)]	*Z/H*	*P*
Age (years)	≤ 44	5 (2.7)	25.00 (21.00, 31.00)	0.997	0.607
	45–59	33 (18.0)	24.00 (21.50, 30.00)		
	≥ 60	145 (79.2)	26.00 (21.00, 31.00)	3.490	3.490
Sex	Male	119 (65.0)	25.00 (21.00, 30.00)	−1.968	0.062
	Female	64 (35.0)	27.00 (23.00, 32.75)		
BMI (kg/m^2^)	＜18.5	6 (3.3)	25.00 (20.50, 33.25)	0.184	0.912
	18.5–23.9	117 (63.9)	25.00 (25.00, 31.00)		
	＞23.9	60 (32.8)	25.50 (23.00, 31.00)		
Marital status	Single	2 (1.1)	26.50 (24.00, -)	5.379	0.146
	Married	149 (81.4)	25.00 (21.00, 31.00)		
	Divorced	11 (6.0)	28.00 (23.00, 31.00)		
	Widowed	21 (11.5)	29.00 (25.00, 32.50)		
Form of residence	Lonely	25 (13.7)	29.00 (24.50, 33.00)	6.916	0.031
	With family	81 (44.3)	21.00 (25.00, 31.00)		
	With partner	77 (42.1)	25.00 (21.00, 30.00)		
Educational level	Non-literate	30 (16.4)	28.50 (24.75, 33.00)	4.486	0.214
	Junior high school and below	94 (51.4)	25.00 (21.00, 31.00)		
	High school/technical secondary school	38 (20.8)	25.00 (21.75, 31.00)		
	University/college degree and above	21 (11.5)	26.00 (21.00, 28.50)		
Monthly per capita household income (RMB)	Below 3000	9 (4.9)	23.00 (18.50, 31.00)	6.926	0.074
	3000–5000	52 (28.4)	28.00 (23.00, 31.75)		
	5000–8000	64 (35.0)	25.50 (22.00, 32.00)		
	Above 8000	58 (31.7)	23.50 (21.00, 29.00)		
Types of cancer	Gastric cancer	71 (38.8)	24.00 (21.00, 31.00)	−1.159	0.247
	Colorectal cancer	112 (61.2)	26.00 (22.00, 31.00)		

BMI, body mass index.

### Gastrointestinal cancer survivors' perceptions of social support, social alienation, and type D personality score

Gastrointestinal cancer survivors had a mean total score of 60.58 ​± ​10.94 for perceived social support, 36.25 ​± ​4.71 for social isolation, and 26.26 ​± ​5.84 for type D personality. The scores for each dimension are shown in Table 2.

**Table 2:** Gastrointestinal cancer survivors' perceptions of social support, social alienation, and type D personality scores (*N* ​= ​183).

Items	Entry	Scoring range	Score (Mean ​± ​SD)
Perceived social support	12	12–84	60.58 ​± ​10.94
Family support	4	4–28	20.87 ​± ​3.77
Friends support	4	4–28	18.87 ​± ​4.24
Support from others	4	4–28	20.85 ​± ​3.78
Social isolation	15	15–60	36.25 ​± ​4.71
Sense of others' isolation	5	5–20	12.57 ​± ​2.24
Sense of doubt	4	4–16	9.86 ​± ​1.47
Sense of self-isolation	3	3–12	7.37 ​± ​1.38
Sense of meaninglessness	3	3–12	6.45 ​± ​0.92
Type D personality	14	0–56	26.26 ​± ​5.84
Social inhibition	7	0–28	13.74 ​± ​3.22
Negative affectivity	7	0–28	12.51 ​± ​3.15

### Correlation analyses of perceived social support, social isolation, and type D personality in gastrointestinal cancer survivors

The results of Spearman's correlation analysis are shown in Table 3. Perceived social support was negatively correlated with social isolation and type D personality (*r* ​= ​−0.593, −0.396, both *P* ​< ​0.001). Social isolation was positively correlated with type D personality (*r* ​= ​0.564, *P* ​< ​0.001).

**Table 3:** Correlation analyses of perceived social support, social isolation, and type D personality in gastrointestinal cancer survivors (*N* ​= ​183; *r*).

Items	Perceived social support				Social isolation					Type D personality		
	Total score	Family support	Friends support	Support from others	Total score	Sense of others' isolation	Sense of doubt	Sense of self-isolation	Sense of meaninglessness	Total score	Social inhibition	Negative affectivity
Total score of perceived social support	1	–	–	–	–	–	–	–	–	–	–	–
Family support	0.945^∗∗^	1	–	–	–	–	–	–	–	–	–	–
Friends support	0.894^∗∗^	0.738^∗∗^	1	–	–	–	–	–	–	–	–	–
Support from others	0.957^∗∗^	0.923^∗∗^	0.800^∗∗^	1	–	–	–	–	–	–	–	–
Total score of social isolation	−0.593^∗∗^	−0.529^∗∗^	−0.599^∗∗^	−0.543^∗∗^	1	–	–	–	–	–	–	–
Sense of others' isolation	−0.533^∗∗^	−0.460^∗∗^	−0.565^∗∗^	−0.470^∗∗^	0.915^∗∗^	1	–	–	–	–	–	–
Sense of doubt	−0.323^∗∗^	−0.290^∗∗^	−0.352^∗∗^	−0.285^∗∗^	0.757^∗∗^	0.602^∗∗^	1	–	–	–	–	–
Sense of self-isolation	−0.526^∗∗^	−0.481^∗∗^	−0.481^∗∗^	−0.513^∗∗^	0.719^∗∗^	0.563^∗∗^	0.334^∗∗^	1	–	–	–	–
Sense of meaninglessness	−0.398^∗∗^	−0.387^∗∗^	−0.352^∗∗^	−0.375^∗∗^	0.580^∗∗^	0.415^∗∗^	0.354^∗∗^	0.302^∗∗^	1	–	–	–
Total score of type D personality	−0.396^∗∗^	−0.300^∗∗^	−0.502^∗∗^	−0.351^∗∗^	0.564^∗∗^	0.576^∗∗^	0.370^∗∗^	0.410^∗∗^	0.287^∗∗^	1	–	–
Social inhibition	−0.369^∗∗^	−0.275^∗∗^	−0.475^∗∗^	−0.319^∗∗^	0.528^∗∗^	0.540^∗∗^	0.339^∗∗^	0.418^∗∗^	0.230^∗∗^	0.917^∗∗^	1	–
Negative affectivity	−0.350^∗∗^	−0.264^∗∗^	−0.437^∗∗^	−0.321^∗∗^	0.499^∗∗^	0.503^∗∗^	0.314^∗∗^	0.345^∗∗^	0.308^∗∗^	0.921^∗∗^	0.706^∗∗^	1

∗∗*P* ​< ​0.01.

### The mediating effect of social isolation between perceived social support and type D personality in gastrointestinal cancer survivors

Structural equation modeling of the mediating effect of social isolation between perceived social support and type D personality was established based on Spearman correlation analysis. Because of the small sample size, we employed partial least squares structural equation modeling (PLS-SEM) to confirm the mediating effect. PLS-SEM does not strictly require the data to be normally distributed, and statistically significant effects can be obtained when the sample size is greater than 100. It is well suited for predictive and theory development studies.^35^

### Measurement model evaluation

**Fig. 1:**
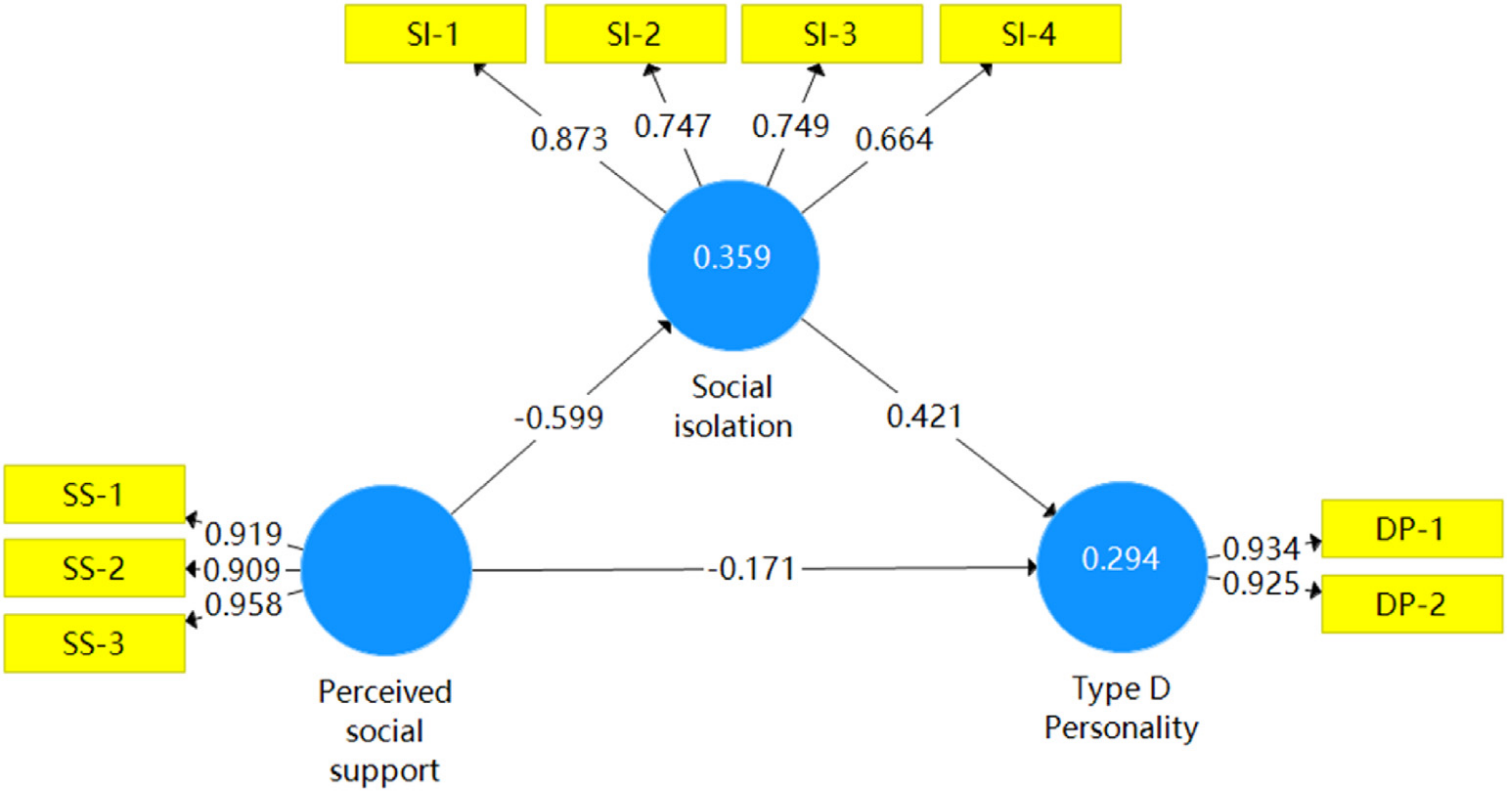
Mediated effects model.

The factor loading of the sense of meaninglessness on the sense of social isolation in the model was 0.664, which is less than 0.7. Considering that the AVE of the model was greater than 0.5 and that 0.664 was already close to 0.7, the apparent variable of the sense of meaninglessness was retained in this study. The model demonstrated good reliability and validity, as shown in Tables 4 and 5. The values between the latent variables and the manifest variables indicate the factor loadings and the values between the latent variables indicate the total effects. The model is shown in Fig. 1.

**Table 4:** Reliability and convergent validity evaluation of the mediated effects model.

Latent variable	Phanero-variable	Factor loading	CA	CR	AVE
Perceived social support	Family support	0.919	0.843	0.927	0.864
	Friends support	0.909			
	Support from others	0.958			
Social isolation	Sense of others' isolation	0.873	0.921	0.950	0.863
	Sense of doubt	0.747			
	Sense of self-isolation	0.749			
	Sense of meaninglessness	0.664			
Type D personality	Social inhibition	0.934	0.756	0.846	0.581
	Negative affectivity	0.925			

CA, Cronbachs Alpha; CR, Composite Reliability; AVE, Average Variance Extracted.

**Table 5:** Evaluation of the differential validity of the mediated effects model (*N* ​= ​183).

Latent variable	Type D personality	Perceived social support	Social isolation
Type D personality	0.930		
Perceived social support	−0.424	0.929	
Social isolation	0.524	−0.599	0.762

### Evaluation of the structural model

In this study, the Bootstrap sampling number was set to 5000. As shown in the table, At a significance level of *P* ​< ​0.05, the T-value is larger than 1.96, and at a level of *P* ​< ​0.001, it is greater than 3.29. The loading coefficients are all over 0.5, indicating that the measurement model is well-fitted and that the latent variables are well reflected by the observed variables, as detailed in Table 6. The *R*^2^ for social isolation in the model is 0.359, and the *R*^2^ for type D personality is 0.294, both of which show that the model satisfies the requirements because they are both higher than 0.019.

**Table 6:** Bootstrap test results of loading coefficients of the constructed model (*N* ​= ​183).

	Original sample	Sample mean	Standard deviation	T-Value (|O/STDEV|)	*P*-value
PSS→TDP	−0.171	−0.167	0.077	2.213	0.027
PSS→SI	−0.599	−0.604	0.052	11.547	0.000
SI→TDP	0.421	0.428	0.075	5.600	0.000

PSS, Perceived social support; SI, Social isolation; TDP, Type D personality.

### Mediating effects

On the main effects test, the effect of perceived social support on social isolation was −0.599, the effect of social isolation on type D personality was 0.421, and the total effect of perceived social support on type D personality was −0.424. On the mediated effects test, the indirect path coefficient of perceived social support on type D personality was −0.599 ​× ​0.421 ​= ​−0.253. The magnitude of the mediating effect can be assessed using the explained variance ratio (VAF), where VAF = (indirect path coefficient / total effect path coefficient) ​× ​100%. A VAF value of < 20% represents no mediating effect, 20%–80% represents a partially mediated effect, and > 80% represents a fully mediated effect.^36^ The VAF value in this study was (−0.253 / −0.424) ​× ​100% ​= ​59.67%, indicating a partially mediated effect.

## Discussion

### Discussion based on theoretical models

Based on WHITE's heuristic cognitive-behavioral model of emotional cognition theory^29^ we proposed the hypothesis that social isolation in gastrointestinal cancer survivors apprehending the ability to mediate between perceived social support and type D personality, aiming to provide a rationale for reducing the incidence of type D personality in gastrointestinal cancer survivors. Ultimately, our study also verified this hypothesis and drew the following conclusions. Gastrointestinal cancer survivors during radiotherapy had a moderate level of social isolation, an overrepresentation of type D personality, and a moderate level of perceptual social support.

The results of this study showed that the social isolation score of gastrointestinal cancer survivors was (36.25 ​± ​4.71), which was moderately high compared with the total scale score of 60. It was lower than the score of Zhang et al.^37^ for recovering breast cancer patients (41.23 ​± ​10.17) and Chen et al.^38^ for colorectal cancer survivors (45.55 ​± ​5.72). The reason for the difference may be related to variations in sample selection and the different stages of the disease.

The higher mean scores of the dimensions of social isolation are the sense of isolation from others and the feeling of suspicion. This could be due to the inclusion of the research subjects in this study in the radiotherapy period. The painful experiences suffered by the patients during treatment, such as nausea and vomiting, hair loss, fatigue, and other symptomatic disturbances, as well as negative emotions such as stress, anxiety, and depression,^39^ led to an increased sense of isolation from others.^40^ Additionally, the research subjects of this investigation included patients with intestinal stoma, and the physiological and pathological changes are more likely to cause changes in psychological and social functioning.^41^

The patients' inability to control their bowel movements,^42^ the need to carry an ostomy bag for long periods, the presence of fecal leakage odor, and the fear of being negatively judged by their close family and friends exacerbated negative emotions, leading to higher scores of skepticism.

The study findings revealed a perceived social support score of (60.58 ​± ​10.94) among gastrointestinal cancer survivors, which was higher than the results from Ju et al.,^43^ on perceived social support in middle-aged and young stroke patients. This variation can be attributed to the fact that most gastrointestinal cancer survivors in this study were elderly, a group often receiving more attention from relatives, friends, and others compared to younger individuals.^44^ Additionally, this group might benefit from an established extended care platform, which provides disease management knowledge and professional Q&A from the healthcare team, resulting in better social support and an increased sense of care and respect.

The detection rate of type D personality among gastrointestinal cancer survivors was 81.4%, which is higher than the 33.07% reported by Cao et al.^45^ This variation suggests that type D personality, as a personality subtype, shows considerable variability in detection rates across studies. This may be related to the stage of the disease, age group, and geographical location of the study population. During the chemoradiotherapy period, although chemotherapy prolongs the life of patients to a certain extent, it kills cancer cells and also causes a series of reactions such as fatigue, hair loss, nausea, vomiting, myelosuppression, fear, depression, etc., which is a powerful physical and psychological double stress that causes patients to have a serious sense of shame and inferiority, and therefore, may lead to a high detection rate. In addition, the age distribution of this study was mostly middle-aged and old people. Zhang et al. showed that type D personality accounted for a higher percentage of gastric cancer survivors aged 65–80 years,^18^ while the average age in this study was (67.89 ​± ​9.73) years old on average, so it was considered that the high detection rate might be related to the age of the included population. Finally, most of the current studies on the detection rate of type D personality in cancer survivors are foreign studies, few of which are available within China, and the sample size of the present study was relatively small, which may have some bias. In the future, multi-center and large samples can be adopted to validate the detection rate.

In our study, we found that perceived social support is negatively correlated with social isolation, which is consistent with the findings of HU et al.^46^ The results of a cross-sectional study by Jia et al. examining older adults in China suggest that navigating social support may buffer the negative effects of loneliness on social isolation.^47^ This negative relationship was also confirmed in a study of breast cancer survivors,^9^ which further illustrated that perceived social support can provide individuals with emotional comfort, practical help, and informational support, thereby reducing their sense of social isolation. When individuals can receive understanding, care, and support from others when facing difficulties and challenges, it will enhance their connection to society and sense of belonging, and reduce the degree of social isolation. In addition, the related research highlighted that perceived social support can not only directly reduce the sense of social isolation, but also indirectly reduce the occurrence of social isolation by improving the individual's psychological state and coping ability.^48^^,^^49^ In summary, the negative correlation between comprehension of social support and social isolation has been verified in several studies, which is of great significance for understanding and intervening in the phenomenon of social isolation.

Therefore, it is recommended that caregivers actively cultivate robust social support networks, including compassion groups, peer support initiatives, and strengthened family connections. These networks can play a pivotal role in assisting gastrointestinal cancer survivors in seamlessly reintegrating into social life. By fostering these supportive environments, caregivers can enhance the survivors' emotional resilience, alleviate feelings of isolation, and promote a greater sense of belonging within their communities. It is suggested that the community form a compassionate group^50^ to bring together healthcare workers, neighbors, and friends to form a support network in the community to provide necessary support and help for gastrointestinal cancer survivors. Another patient-centered group activity is regularly held to provide gastrointestinal cancer survivors with opportunities for peer-to-peer exchanges, where patients with colostomy can communicate with each other about stoma care, give peer support and encouragement, improve patients' social motivation, and increase their sense of social presence. Hospitals can consider family-centered interventions, such as binary coping of husband and wife, family empowerment, and organization of family meetings, to help gastrointestinal cancer survivors establish a good family bond, ease the communication gap between patients and their families, eliminate the patient's worries and concerns about the increased financial burden of stoma surgery on the family, enhance family care, and give patients more emotional support and value affirmation from family members.^51^ In addition, for intestinal stoma patients, healthcare professionals can use social media to establish the structure and function of social contact, and hospitals can rely on social network platforms to organize patients to participate in online forums for stomas, to share information, or carry out continuity of care to patients, to recommend available social support resources to patients, and to encourage them to take the initiative in expanding their social interactions.^52^

In addition, perceived social support is negatively correlated with type D personality, i.e., cancer survivors with higher levels of perceived social support will also have improved negative affect and social inhibition in type D personality. Wang et al. found that perceived family support had a moderating effect between social isolation and levels of depression in college freshmen.^53^ Type D personality is a stable personality trait and changing it can be a long and difficult process, but it is easier to get support from family. This is because individuals with type D personality are more likely to feel depressed, and if their perceived level of comprehension of family support is low, it increases the risk of depression occurring.^54^ Xu et al.^55^ showed that in patients with hematologic malignancies, type D personality scores were significantly and negatively correlated with perceived social support. Social support can alleviate the psychological pain of cancer patients, and a supportive social network can help individuals cope with various stressful events and reduce their physical and psychological harm,^56^ type D personality participants in the coronary artery disease population scored significantly lower on the three dimensions of perceived social support (family, friends, and others).^57^ All of the above studies are consistent with the results of the present study. Therefore, it is crucial to bolster the perceived social support for gastrointestinal cancer survivors. These individuals require both emotional and material assistance from family members, healthcare professionals, and their communities.

Social isolation in gastrointestinal cancer survivors is positively correlated with type D personality, and patients with type D personality tend to have more negative emotions, such as anxiety and depression,^23^ which have a direct impact on psychological stress; in addition, patients with type D personality have a large degree of social inhibition, are afraid of communicating and interacting with others, and have a high sense of social detachment.^58^ Research has shown that type D personality is positively correlated with social isolation, i.e., individuals with type D personality are more likely to be socially isolated. Individuals with type D personality are more inclined to inhibit self-expression in social interactions, which leads to less contact with others and less social engagement, and thus to social isolation.^23^ The results of this study indicate that individuals with type D personality are more likely to be socially isolated. Consequently, nursing staff can employ the Progressive Focused Interviewing Program to intervene and mitigate the negative emotions associated with type D personality. This approach involves guiding patients to articulate their inner thoughts, encouraging self-reflection, facilitating stress release, promoting active participation in social activities, enhancing their sense of self-worth, and alleviating feelings of social isolation.

Social isolation partially mediated the relationship between perceived social support and type D personality (VAF ​= ​59.67%). That is, perceptions of social support can directly influence gastrointestinal cancer survivors' type D personality, as well as through social isolation. On an individual level, when gastrointestinal cancer survivors are socially isolated, their social interactions with others decrease and their emotional connections become weak. This sense of social isolation affects the individual's appreciation of social support. Originally, one may be able to perceive the care and support from family, friends, coworkers, etc., but in a state of social isolation, the individual may become slow to perceive this support. For example, in daily life, a person who has little communication with others and keeps to himself or herself may not be able to appreciate the power of social support in a timely and accurate manner when others reach out to him or her or express their concern for him or her, thus affecting the degree of perception of social support.

Individuals with type D personality are characterized by negative affect and social inhibition. This personality trait may be further exacerbated by social isolation, as those with type D personalities are inherently inclined to suppress their emotions and limit contact with others. This tendency is intensified in socially isolated environments. Social isolation serves as a critical link between perceived social support and type D personality. On one hand, social isolation hinders individuals with type D personality from experiencing social support, deepening their sense of loneliness and negative emotions; on the other hand, the absence of social support may reinforce their type D traits, creating a vicious cycle.

The WHITE heuristic cognitive-behavioral model^29^ suggests that practical material and emotional support from family and others can reduce negative emotions in patients by mitigating social isolation. Support from family, friends, and social organizations helps patients adapt to disease symptoms and social disruptions, encouraging them to seek help and engage more actively in social activities.^59^ This increased social interaction can reduce social isolation and foster more optimistic emotional experiences, decreasing negative emotions like anxiety and depression, and preventing the formation of a type D personality. Thus, gastrointestinal cancer survivors with type D personalities are relieved of negative emotions.

To address this issue, clinical caregivers can effectively employ Progressive Focused Interviewing (PFI), Positive Mindfulness Stress Reduction Therapy, and Theory of Wisdom psychotherapy to mitigate social isolation among gastrointestinal cancer survivors. PFI, a transformative psychological intervention, begins by exploring the patient's daily life and behaviors. By initially establishing a close, trusting relationship, the interviewer can then gradually guide the conversation toward more targeted areas. This approach allows for deeper exploration of the patient's inner world, stimulating their self-potential and enhancing psychological flexibility, ultimately fostering a greater sense of connection and emotional resilience.^60^ It has been shown that progressive-focused interviews with patients with colostomy can reduce their sense of shame, change their coping styles, and enable them to return to their families and society by identifying the patients' psychological problems and providing positive guidance. Positive thinking stress reduction therapy can be carried out in the hospital, including positive thinking meditation, body scanning, positive thinking walking, positive thinking yoga, and other relaxation techniques to awaken the inner focus of patients with enterostomies, improve physical and mental regulation, and help patients integrate into the society in a good state of mind.^61^ Wisdom theory psychotherapy refers to the establishment of a harmonious relationship between medical workers and patients with the function of counseling, based on which patients with enterostomies can be guided to participate in group activities and be given group counseling to improve their postoperative social adaptability and interpersonal communication, and to guide the patients to face up to themselves and their illnesses, and to form an open and accepting attitude to expand the scope of social interaction.^51^ Therefore, it is recommended that healthcare workers adopt the above psychological interventions to enhance the perceived social support of gastrointestinal cancer survivors, and through these psychological interventions to directly reduce the patients' sense of social isolation, and to guide people with type D personality to reduce their negative emotions and increase their social interactions.

### Implications for nursing practice and research

It is recommended that nurses should improve the way they educate gastrointestinal cancer survivors about managing their symptoms while receiving radiation therapy. They should encourage physical exercise, improve self-management skills, and help reduce psychological burdens. Additionally, family members, especially spouses, should strengthen communication with patients, providing ample support, attention, understanding, and love. Encouraging open emotional expression and fostering mutual understanding is crucial.^62^

Moreover, it is important to prioritize the physical and mental well-being of gastrointestinal cancer survivors in different sectors. This includes ensuring non-discrimination in job positions and fair treatment to facilitate their return to normal work and life. Interventions to address social isolation, such as positive psychology group therapy, stress reduction techniques, and commitment and acceptance therapy, should be considered to enhance patients' inner strength and guide them in understanding and coping with social isolation.

Furthermore, promoting community activities to encourage interpersonal and social exchanges is beneficial. Establishing support groups and other initiatives can increase patients' sense of psychological support and further facilitate their smooth reintegration into society.

### Limitations

There are several limitations in this study, mainly in: (1) Using convenience sampling, and only gastrointestinal cancer survivors from a tertiary hospital in Jiangsu Province were selected as the study population. The restricted study area may have compromised the sample's representativeness. In the future, employing a multi-stage random sampling method could enhance the study by including multiple areas and increasing the sample size. (2) In terms of research methodology, the use of a cross-sectional questionnaire may introduce self-reporting bias as patients complete the survey. In addition, cross-sectional surveys cannot establish causal relationships between variables. In the future, more research methods can be used to consolidate and explore the conclusions.

## Conclusions

Among gastrointestinal cancer survivors experiencing chemoradiotherapy, social isolation was moderately high, type D personality was overrepresented, and perceived social support was moderate. Type D personality was positively correlated with social isolation and negatively correlated with perceived social support. The association between type D personality and perceived social support was partially mediated by social isolation. This suggests that caregivers need to use relevant interventions to alleviate cancer survivors' sense of social isolation, and should also focus on constructing a social support system to enhance their perceived social support to reduce the negative emotions of type D personality in gastrointestinal cancer survivors and increase their social interaction.

## CRediT authorship contribution statement

**Yuqiu Zhao**: Conceptualization; Methodology; Data curation; Formal analysis; Original draft. **Yeming Xu**: Methodology; Data curation; Formal analysis. **Tianxiu Wang**: Collect and verify data. **Mingbo Hua**: Collect and verify data. **Shantanu Baral**: Review & edit. **Qiannan Sun**: Supervision; Review & editing. **Daorong Wang**: Supervision; Review & editing. All authors had full access to all the data in the study, and the corresponding author had final responsibility for the decision to submit for publication. The corresponding author attests that all listed authors meet authorship criteria and that no others meeting the criteria have been omitted.

## Ethics statement

This study has been approved by the Ethics Committee of Yangzhou University School of Nursing School of Public Health (IRB No. YZUHL20230078). All participants provided written informed consent.

## Funding

This work was supported by Yangzhou Innovation Capacity Building Program Key Laboratory of Basic and Clinical Transformation of Digestive Diseases/Metabolism (Grant No. YZ2020159). The funders had no role in considering the study design or in the collection, analysis, interpretation of data, writing of the report, or decision to submit the article for publication.

## Data availability statement

The data that support the findings of this study are available from the corresponding author, Daorong Wang, upon reasonable request.

## Declaration of generative AI and AI-assisted technologies in the writing process

No AI tools/services were used during the preparation of this work.

## Declaration of competing interest

The authors declare no conflict of interest.
